# COVID-19-Induced Cardiac Tamponade: A Case Study and a Literature Review

**DOI:** 10.7759/cureus.42427

**Published:** 2023-07-25

**Authors:** Rishabh Mishra, Swati Jain, Mohammed Shaban, Giancarlo Acosta, Miguel A Rodriguez Guerra

**Affiliations:** 1 Hospital Medicine, Montefiore Medical Center, Wakefield Campus, Bronx, USA; 2 Nephrology, Montefiore Medical Center, Wakefield Campus, Bronx, USA; 3 Internal Medicine, BronxCare Health System, Bronx, USA; 4 Cardiology, Marshall University, Huntington, USA; 5 Medicine, Montefiore Medical Center, Albert Einstein College of Medicine, Bronx, USA

**Keywords:** sars-cov-2, covid cardiac tamponade, covid pericardial effusion, pericardial infusion, cardiac tamponade

## Abstract

COVID-19 presentation is heterogeneous. As a viral illness, it could cause pericardial effusion leading to cardiac tamponade. We present a patient coursing with this viral illness that was found to have cardiac tamponade. We report a case of a 79-year-old female who presented with shortness of breath and dry cough for one week and resulted positive for severe acute respiratory syndrome coronavirus 2 (SARS-CoV-2). Her initial chest X-ray showed a bottle-shaped heart. Computed chest tomography showed pericardial effusion, and an echocardiogram confirmed moderated pericardial effusion with signs of tamponade. He improved with conservative therapy with colchicine, ibuprofen, cefepime, dexamethasone, dolutegravir, and apixaban for pulmonary emboli. An early approach in cardiac tamponade induced by COVID-19 is crucial to promptly address an aggressive directed therapy, avoiding potential complications or unnecessary procedures.

## Introduction

Pericardial effusion could be present in multiple conditions as inflammatory disorders, renal disease, malignancy, trauma, certain medications, and autoimmune disorders. Viral, bacterial, or fungal infections can cause infectious pericarditis such as Coxsackievirus, Epstein-Barr virus, and influenza viruses. It is crucial to identify and treat the underlying infectious cause to manage pericardial effusion effectively. COVID-19 can have variable presentations; however, prior to the era of COVID-19 vaccinations, there was not enough data establishing the presence of pericardial effusion with tamponade and its therapy [1-5]. This is the case of a patient who was found to have cardiac tamponade induced by the first wave of COVID-19. Ultimately, the pericardial effusion has improved with prompt supportive care and integrase inhibitor therapy.

## Case presentation

**Figure 1:**
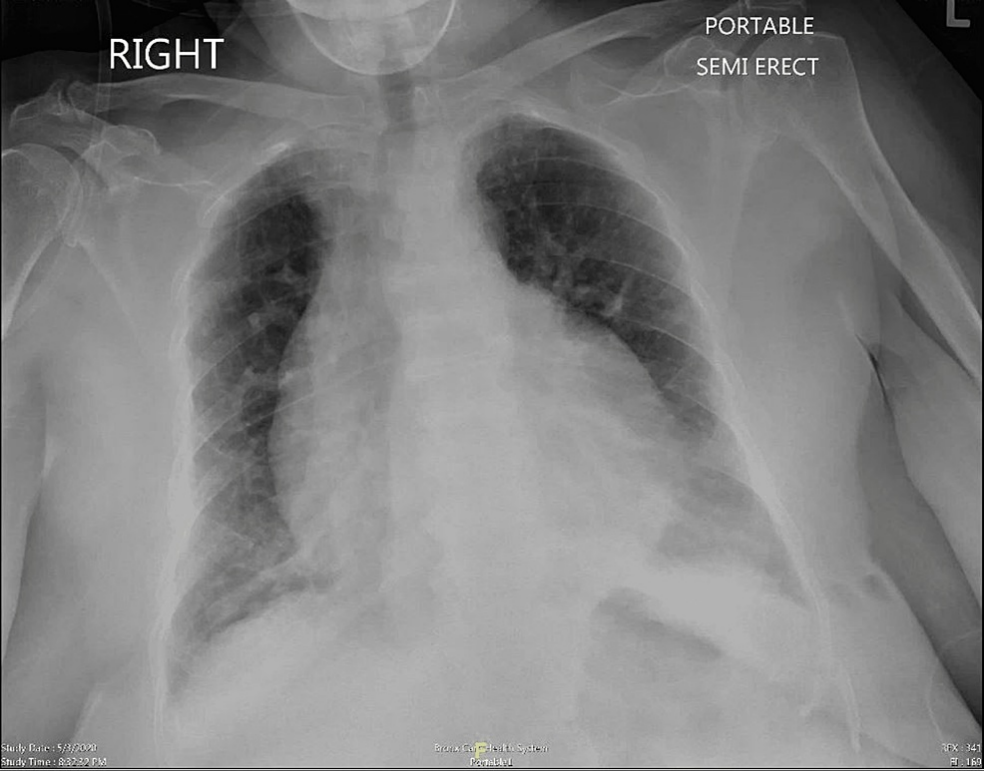
Chest X-ray with a bottle-shaped cardiac silhouette

This is the case of a 79-year-old female with a past medical history of asthma, hypertension, osteoporosis, and diabetes; she presented to our emergency department with persistent shortness of breath associated with a dry cough for one week. The patient denied prior episodes, trauma, fever, or chills. The physical examination was unremarkable. COVID-PCR was positive, and chest X-ray (CXR) showed left infiltrates and a bottle-shaped cardiac silhouette (Figure 1).

**Figure 2:**
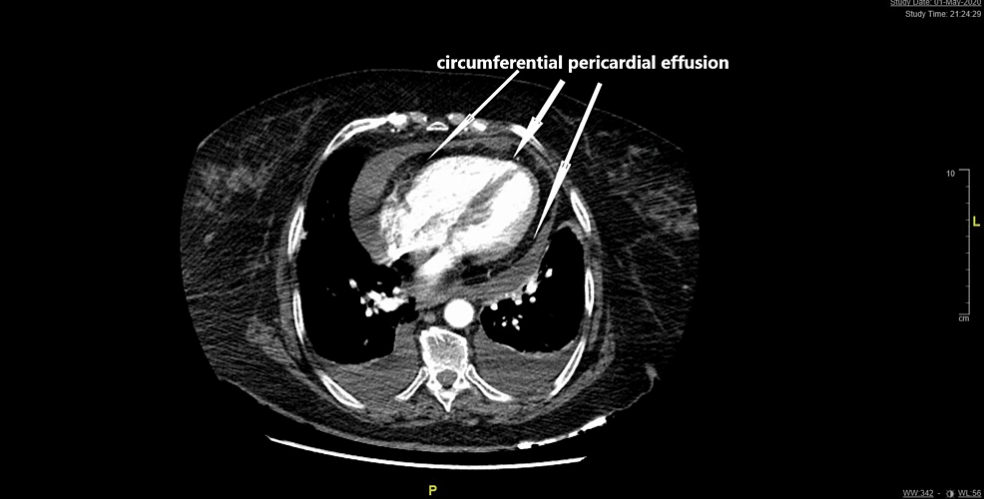
CT chest showing moderate circumferential pericardial effusion

The patient presented records of prior normal CXR prior to her presentation. Computed chest tomography showed small pulmonary emboli, pneumonia, and moderate pericardial effusion (Figure 2).

**Figure 3:**
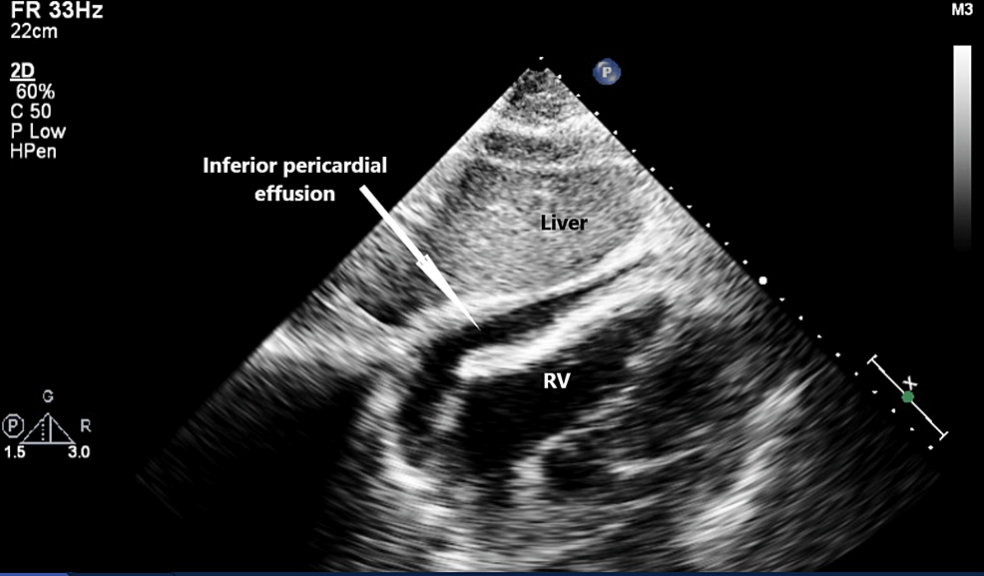
Inferior pericardial effusion (subcostal view) RV, right ventricle.

**Figure 4:**
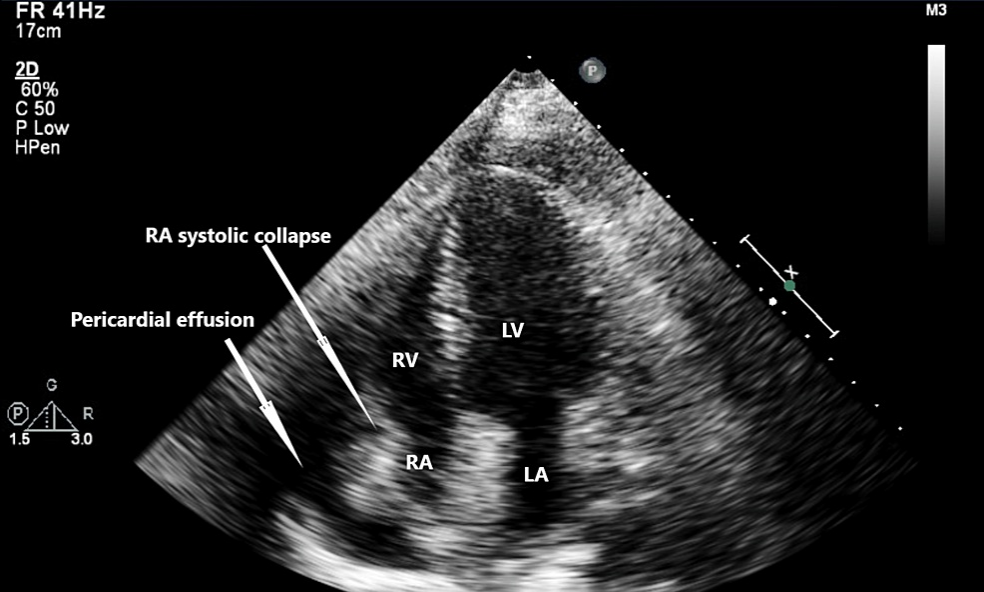
Right atrial collapse secondary to pericardial effusion RV, right ventricle; RA, right atrium; LV, left ventricle; LA, left atrium.

The echocardiogram confirmed the moderate pericardial effusion with right atrial systolic collapse concerning for early tamponade (Figures 3, 4).

**Figure 5:**
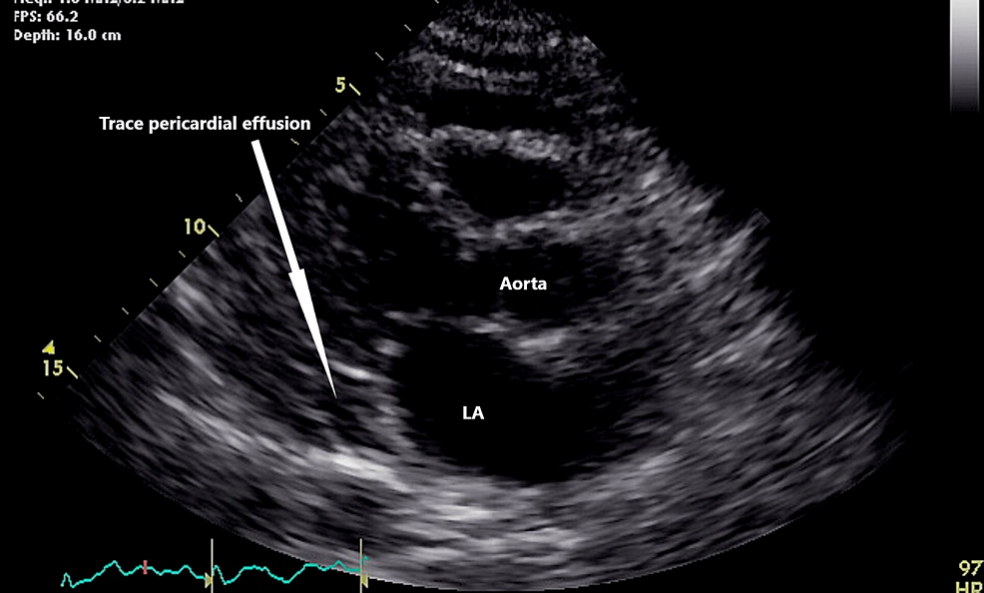
Trace pericardial effusion after treatment with colchicine and steroids LA, left atrium.

The patient was treated with colchicine, ibuprofen, cefepime, dexamethasone, dolutegravir, and apixaban. Before discharge, the echocardiogram was repeated and showed a trace effusion without tamponade signs (Figure 5).

## Discussion

Pericardial effusion can be caused by infections, inflammation, and autoimmune disorders; in some cases, the cause cannot be identified. The symptoms of this condition may depend on its severity [6]. Although mild cases may be asymptomatic, patients with moderate effusions can present with chest pain, shortness of breath, palpitations, cough, and fatigue [7]. However, severe cases may lead to cardiac tamponade, shock, and sudden cardiac death [8].

COVID-19 is a respiratory illness caused by the SARS-CoV-2 virus [9]. This entity is known to affect multiple organ systems, and there are reports of inflammatory consequences affecting the heart [10].

COVID-19 can induce pericardial effusion, possibly due to an inflammatory reaction in the pericardium, but its mechanism is not entirely understood [11]. If pericardial effusion is suspected in a person with COVID-19, imaging, including echocardiography, must be performed to evaluate the severity [12]. Its management typically involves supportive care. Severe cases lead to cardiac tamponade, where prompt medical attention is essential; it requires continuous hemodynamic monitoring, and if the patient has severe symptoms or instability, then pericardiocentesis or pericardial window surgery may be required [13,14].

The supportive therapy on pericardial effusion includes anti-inflammatories (non-steroidal anti-inflammatory drugs [NSAIDs], colchicine), steroids, uricosuric agents, and antivirals [15,16]. Corticosteroids have potent anti-inflammatory properties reducing the inflammation of the pericardium, alleviating the symptoms, improving the effusion, and preventing further complications [17,18]. Integrase inhibitors are antiretroviral medications that are not typically used for pericardial effusion. However, this drug may target the virus's replication cycle and potentially benefit in terms of lymphocyte count [19].

The exact mechanisms for COVID-19-induced cardiac tamponade are not fully understood; its initial management typically involves non-invasive supportive care [20]. However, other approaches may be required for patients with severe symptoms or instability, such as pericardiocentesis or pericardial window surgery.

## Conclusions

Pericardial effusion induced by COVID-19 inflammatory syndrome could lead to cardiac tamponade. Our case is an interesting and unique case that highlights the effectiveness of anti-inflammatories, steroids, and integrase inhibitors. Early diagnosis and an aggressive approach could be the most crucial measures for these patients.
